# Bioengineered Tumor‐Derived Extracellular Vehicles Suppressed Colorectal Cancer Liver Metastasis and Bevacizumab Resistance

**DOI:** 10.1002/advs.202417714

**Published:** 2025-05-21

**Authors:** Junjiang Wang, Chunsheng Liu, Ping Wang, Zhiyuan Liu, Weixian Hu, Zejian Lv, Chengzhi Huang, Xueqing Yao

**Affiliations:** ^1^ Department of Gastrointestinal Surgery Department of General Surgery Guangdong Provincial People's Hospital Guangdong Academy of Medical Sciences Southern Medical University Guangzhou 510000 China; ^2^ School of Medicine South China University of Technology Guangzhou 510006 China; ^3^ Department of General Surgery Guangdong Provincial People's Hospital Ganzhou Hospital (Ganzhou Municipal Hospital) Ganzhou 341000 China; ^4^ Department of General Surgery Guangdong Provincial People's Hospital Heyuan Hospital Heyuan 517000 China

**Keywords:** colorectal cancer, antiangiogenic therapy, engineered extracellular vehicles, liver metastasis, siRNA delivery

## Abstract

Antiangiogenic therapies, such as bevacizumab, are among the causes of cancer‐related death in patients with colorectal cancer (CRC) with liver metastasis. Delivering siRNAs via primary cell originating from primary cells is a promising method for targeting CRC liver metastasis and drug resistance. Here, it is found that the expression of CCL24 is significantly upregulated in tumor tissues at the CRC liver metastasis site. In addition, CCL24 is significantly upregulated in tumor tissues from bevacizumab‐resistant patients. CCL24 promotes the formation of inflammatory tumor‐associated fibroblast subsets in the CRC liver metastasis microenvironment and induces resistance to bevacizumab therapy. Based on these results, a primary cell‐derived extracellular vehicle delivery system is designed for the simultaneous delivery of siRNAs targeting CCL24 in the tumor microenvironment (TME). Downregulation of CCL24 in the TME by delivering bioengineered extracellular vehicles significantly increased sensitivity to antiangiogenic therapy in a CRC mouse model. A novel therapeutic target is identified for patients with CRC with liver metastasis and suggested a possible therapeutic alternative for patients with CRC with resistance to antiangiogenic therapy and distant metastasis.

## Graphical abstract

The upregulated CCL24 in CRC liver metastasis promotes the formation of inflammatory tumor‐associated fibroblast subsets and induces resistance to bevacizumab therapy. Downregulation of CCL24 significantly increased sensitivity to antiangiogenic therapy in CRC mouse model. Hence, a novel therapeutic target is identified for patients with CRC with liver metastasis and suggested a possible therapeutic alternative for patients with CRC with resistance to antiangiogenic therapy and distant metastasis.

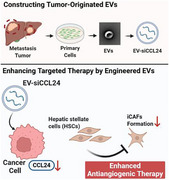

## 1 Introduction

Colorectal cancer (CRC) is the third most common malignant tumor worldwide.^[^ ^1^ ^]^ Liver is one of the most common organs affected by the distant metastases of CRC.^[^ ^2^ ^]^ Specifically, CRC liver metastasis (CRCLM) contributes to more than 75% of cancer‐related mortalities, making CRC the second most fatal malignancy among cancers.^[^ ^3^ ^]^ Synchronous liver metastasis is present in 20–40% of patients with CRC at the time of initial diagnosis.^[^ ^4^ ^]^ However, even if patients with CRC undergo radical surgery, up to 50% eventually develop hepatic metastases.^[^ ^2^, ^3^ ^]^ Radical hepatic tumor resection is the only long‐term survival option for patients with CRCLM; however, more than 80% of patients with CRCLM develop unresectable metastatic tumors and are unable to undergo radical surgery at the time of diagnosis.^[^ ^3^, ^5^ ^]^ Therefore, novel biomarkers and effective targeted or combination therapies to shrink the size of metastatic tumors to allow patients with CRCLM to undergo radical surgery are required.^[^ ^6^ ^]^

The combination of chemotherapy and the anti‐VEGF antibody, bevacizumab (Bev), is the current standard treatment for patients with CRCLM, based on the superior overall survival (OS) improvement reported in randomized clinical trials and clinical guidelines.^[^ ^5^, ^6^, ^7^ ^]^ However, according to clinical observations, the OS improvement in patients with CRCLM is limited and metastasis may eventually occur.^[^ ^8^ ^]^ Moreover, the withdrawal of VEGF from the tumor microenvironment (TME) by antiangiogenic therapy may be strongly associated with increased extracellular matrix deposition, which may enhance the chemotherapy resistance of malignant tumors.^[^ ^8^, ^9^ ^]^ The pathophysiological mechanisms underlying acquired resistance to antiangiogenic therapy have not been fully elucidated. Cancer‐associated fibroblasts (CAFs) are pro‐tumorigenic components of the TME and may play an immunosuppressive role in the secretion of factors that promote tumor invasion.^[^ ^10^ ^]^ CAFs in the CRC liver metastasis TME was mainly categorized as myoblastic CAFs (myCAFs) and inflammatory CAFs (iCAFs).^[^ ^11^ ^]^ High infiltration of iCAFs into the TME may contribute to CRCLM tumor progression and combination therapy resistance via the secretion of immunomodulatory and pro‐tumorigenic factors, such as chemokines and fibroblast growth factors.^[^ ^11^, ^12^ ^]^ The infiltration of myCAFs may enhance tissue stiffness in liver metastases compared with that in primary colorectal tumors, resulting in enhanced angiogenesis and antiangiogenic therapy resistance.^[^ ^13^ ^]^ However, the functional roles of myCAFs and iCAFs remain controversial, as recent studies have reported that myCAFs may inhibit CRCLM progression by secreting type I collagen, which restricts the invasion of tumor cells.^[^ ^14^ ^]^ Chemokines, a group of chemoattractant cytokines, are considered critical factors in regulating iCAF subgroup infiltration into the CRCLM TME. Chemokines are divided into sub‐families according to the domain located in the N‐terminal CC domain. Notably, no single CC chemokine is a well‐recognized prognostic factor in all types of tumors, because any given chemokine has both pro‐ and anti‐cancer properties.^[^ ^15^ ^]^ Eotaxins, including C‐C motif chemokine ligand 11 (CCL11, eotaxin‐1), CCL24 (CCL11, eotaxin‐2), and CCL26 (eotaxin‐3), are potent chemoattractants for eosinophils, which are increased in allergic diseases, such as allergic asthma, allergic rhinitis, and atopic dermatitis.^[^ ^15^ ^]^ However, the influence of eotaxins on CRCLM and the regulation of non‐malignant cells in the TME has not been fully elucidated.

The delivery of siRNAs or non‐coding RNAs may be a promising strategy to target liver metastasis and/or chemoresistance in CRC.^[^ ^16^ ^]^ Limited studies have focused on the moderating aspects of tumor‐related angiogenesis in the TME. Based on our previous Extracelluar vesicles‐based delivery nanocarriers, a safe, highly effective, and ideal biocompatible delivery system that targets the TME should be considered pivotal for patients with CRC. Here, we report novel, patient‐derived, primary cell‐derived extracellular vesicles as non‐coding RNA delivery systems.

Extracellular vesicles are small (40–120 nm) membrane vesicles of endocytic origin that are released into the extracellular environment by various cells.^[^ ^17^ ^]^ The presence of mRNAs, miRNAs, long non‐coding RNAs, or proteins in extracellular vesicles is a possible mechanism for genetic exchange and cell‐to‐cell communication in the TME.^[^ ^18^ ^]^ The natural characteristics of extracellular vehicles facilitate efficient delivery of therapeutic siRNAs across biological barriers and maintain their stability in blood circulation.^[^ ^19^ ^]^ More importantly, the extracellular vehicles may gain tumor‐targeting ability because they are originally derived from the same type of cells and act as a communication tool in the tumor microenvironment.^[^ ^18a^ ^]^ An increasing number of studies have focused on exploiting these features to engineer natural extracellular vehicles for drug or functional nucleic acid delivery for specific diseases. Owing to the higher heterogeneity of tumors among patients, an effective and precision‐guided method for innovative cancer therapy is required. However, the identification of novel therapeutic targets from clinical samples and development of highly effective therapeutic nucleic acid delivery systems using primary cells from patients have not yet been studied and analyzed in detail.

In the present study, we enrolled patients with CRC with recurrent liver metastases, and collected primary and recurrent tumor samples. The potential Bev‐therapy‐related key genes were validated using RNA sequencing. Through bioinformatics analysis, we revealed that higher expression of CCCL24 (eotaxin‐2) might be highly related to tumor metastasis and Bev‐mediated therapy resistance. We further validated these findings using clinical samples. Based on these results and our previous studies, we designed a primary cell‐derived extracellular vehicle delivery system for the simultaneous delivery of siRNAs targeting CCL24 to the TME, with the aim of increasing the sensitivity to targeted therapy reagents. Downregulation of CCL24 in the TME, by delivering bioengineered extracellular vesicles, significantly increased the sensitivity of the CRC mouse model to Bev therapy. We identified a novel therapeutic target for patients with CRC, and suggested a possible therapeutic alternative for those with chemoresistance and distant metastases.

## 2 Results

### 2.1 Upregulation of CCL24 in Tumor Tissues is Associated with Antiangiogenic Therapy in Colorectal Cancer

**Figure 1:**
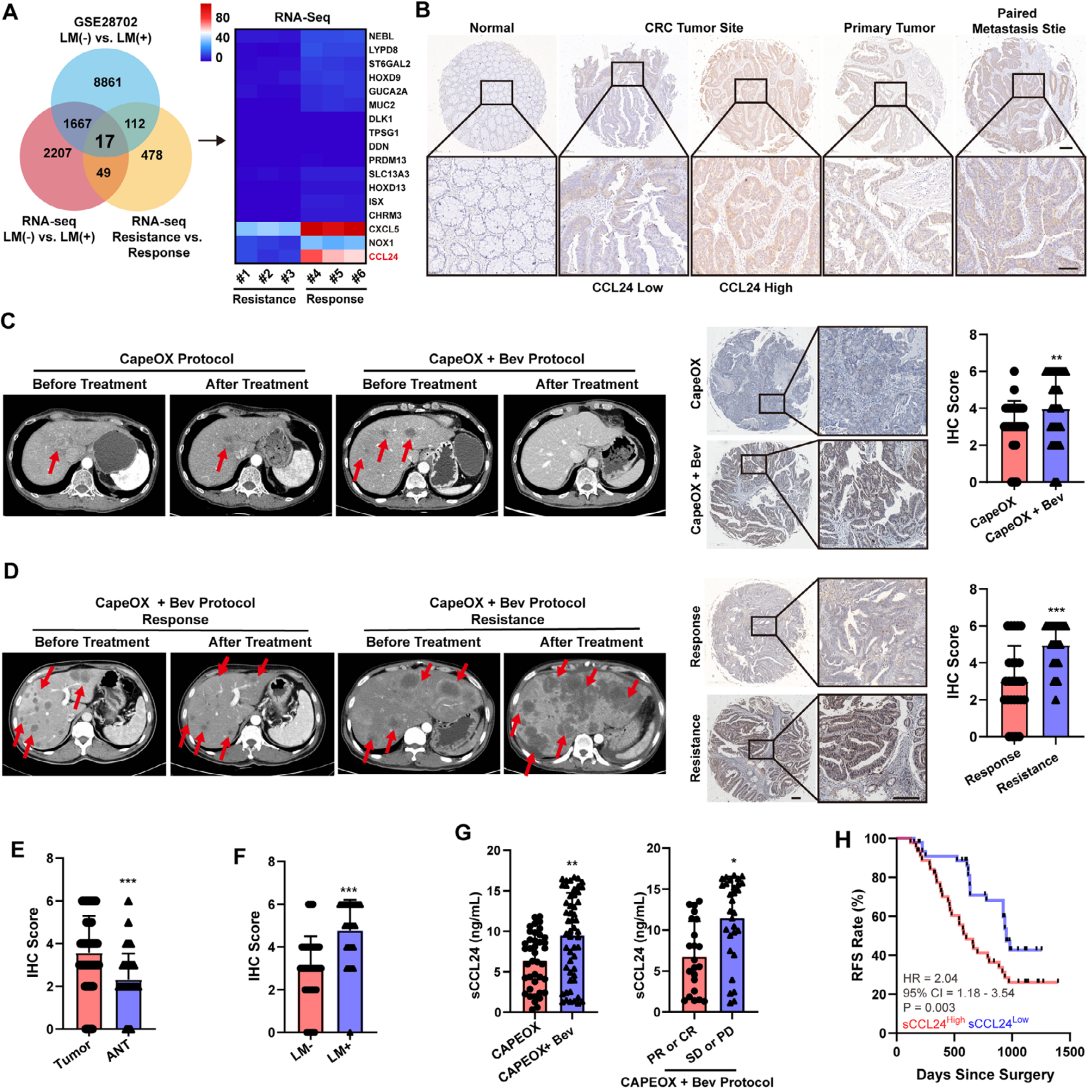
The upregulated CCL24 indicated metastasic CRC Bev therapy resistance and implied poor prognosis. A) Identification of key genes for CRC in cancer metastasis and Bev therapy resistance by RNA‐sequence. The scale bar and the numbers indicates the FPKM of the genes expression. The red color implied the gene was potentially highly expression in the indicated group. The CCL24 mRNA was overexpressed in the metastasis tumor tissue and Bev‐therapy resistance tissue. B) The representative IHC photographs of the included CRC tumor tissues. Scale bar = 600µm for 20× magnification images and 100 µm for 400 × magnification images. C,D) Patients with CRC treated with Bev, whose tumor tissues highly express CCL24. CCL24 is significantly highly expressed in Bev treatment‐resistant tissues; The evaluation of therapy was measured by radiology examination. The red arrows indicate the metastatic tumors in liver. E,F) The IHC score indicated that the CCL24 was overexpressed in metastasic tumor tissue and therapy resistance tumor tissue. G) After Bev treatment, the concentration of seurm CCL24 (sCCL24) in CRC patients was significantly upregulated, and the serum CCL24 content increased in patients resistant to Bev treatment; H) The increase in sCCL24 is an independent risk factor for postoperative recurrence in CRC patients. Survival analysis was calculated using the Kaplan‐Meier method and log‐rank test. The comparison between the other two groups was calculated using an unpaired *t*‐test analysis; ns, not significant; *, *P*<0.05; **, *P*<0.01, ***, *P*<0.001 when compared to the control groups.

We enrolled six patients with CRC at our center and collected surgical specimens, including liver metastasis and primary tumors. The enrolled patients underwent a standard eight‐cycle CapeOx chemotherapy in conjunction with Bev therapy for CRC. Of these, three patients with CRC exhibited resistance to the combination therapy and experienced tumor progression or recurrence, while the remaining patients demonstrated tumor regression. Paired primary and metastatic tumor specimens were collected during surgical resection and RNA sequencing was performed to identify potential genes associated with Bev resistance. The specimen collection protocol is shown in **Figure** **1**A. Fragments Per Kilobase of exon model per Million mapped fragments (FPKM). We examined the differentially expressed genes in primary and chemoresistant metastatic tumors, aligning the bioinformatics results with those from the GSE dataset, (GSE28702, metastasis‐related genes). Bioinformatic and RNA‐seq analyses revealed CCL24 overexpression, which may be closely associated with Bev therapy resistance and liver metastasis (Figure 1A). Therefore, CCL24 was selected for further experiments. As depicted in Figure 1B, the expression of CCL24 was analyzed using immunohistochemistry (IHC). Our findings revealed that CCL24 expression in patients with chemoresistant CRC was markedly elevated compared with that in patients with non‐chemoresistant CRC. Moreover, CCL24 was abundantly expressed at the sites of liver metastasis. Therefore, CCL24 expression may play a role in CRC liver metastasis and therapeutic resistance. To delve deeper into the potential role of CCL24 in antiangiogenic therapy and chemoresistance, we expanded our study to include 92 patients with CRC who received chemotherapy or chemotherapy plus antiangiogenic therapy during their treatment. The CRC cohort included 42 patients with CRC who received standard CapeOx chemotherapy, while the remaining patients underwent a standard eight‐cycle CapeOx chemotherapy in conjunction with Bev therapy. To precisely evaluate the therapeutic effect, we used computed tomography (CT) images to comprehensively evaluate the therapeutic effect in patients with CRC (Figure 1C,D). After analyzing the clinical specimens via IHC staining, patients with CRC receiving Bev treatment had high CCL24 expression in tumor tissues. CCL24 was highly expressed in Bev treatment‐resistant tissues (Figure 1C,D). Therefore, increased expression of CCL24 may be closely related to Bev treatment resistance. In the study cohort, CCL24 expression in the tumor tissues was higher than that in the corresponding adjacent normal tissues. High CCL24 expression has also been observed in tumor tissues of patients with metastatic colorectal cancer (mCRC). Therefore, CCL24 may be involved in tumor progression, although the underlying mechanism remains unclear (Figure 1E,F). In the additional CRC patients cohort, we found that after Bev treatment, the serum CCL24 (sCCL24) concentration in patients was significantly upregulated and serological CCL24 content in patients resistant to Bev treatment was increased. Before radical surgery, the upregulation of sCCL24 in patients with CRC suggests an increased rate of postoperative recurrence. Therefore, the increase in sCCL24 content was an independent risk factor for postoperative recurrence in patients with CRC (HR = 2.04, 95%CI = 1.18–3.54, P = 0.003, Figure 1G,H). Collectively, through the analysis of patient specimens, our results suggest that CCL24 overexpression correlates with distant metastasis and therapy resistance in CRC.

### 2.2 Tumor Derived CCL24 Promotes CRC Distance Metastasis and Antiangiogenic Therapy Resistance In Vivo

Western blotting was performed on CRC cell lines, including SW620, SW480, HT‐29, HCT116, and Caco2. The findings indicated that The HCT116 cell line exhibited high levels of CCL24 chemokine expression, whereas the LOVO cell line demonstrated low CCL24 expression (Figure S1A,B, Supporting Information). To explore this further, we used plasmids and siRNA to overexpress or knockdown CCL24 in both LOVO and HCT116 cell lines. Successful CCL24 knockdown was confirmed using western blotting, PCR, and ELISA (Figure S1C,D, Supporting Information). Furthermore, ELISA of the HCT116 cell culture supernatant yielded comparable results, suggesting that CCL24 may have originated from tumor cells within the TME (Figure S1E, Supporting Information).

**Figure 2:**
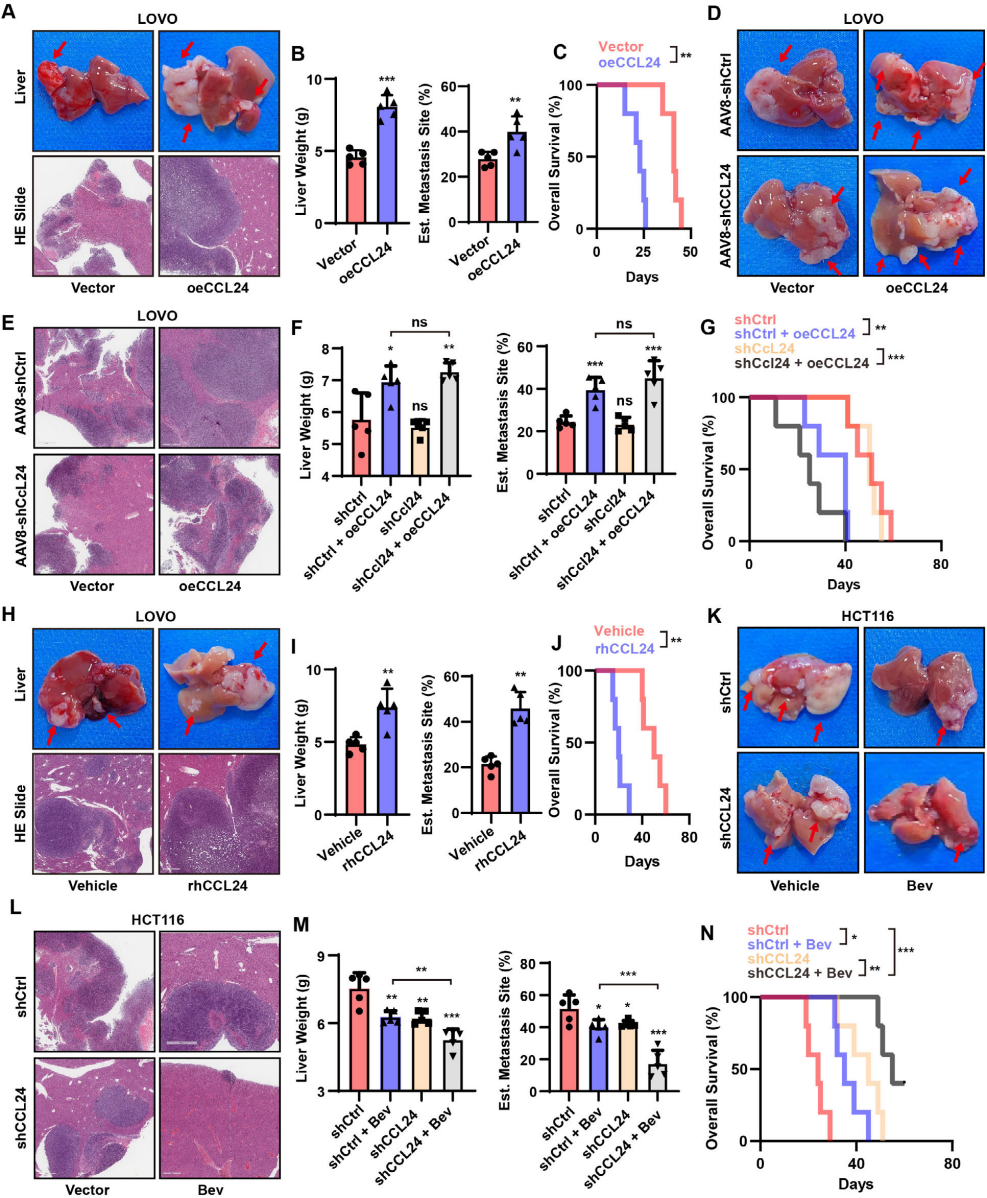
Tumor derived CCL24 promotes CRC distance metastasis and antiangiogenic therapy resistance in vivo. A–C) Overexpression of CCL24 in LOVO cells leads to a significant increase in tumor burden and a decrease in survival in mCRC animal models; D–G) Knocking out exogenous recombinant CCL24 chemokine, overexpression of CCL24 in LOVO cell lines significantly promotes tumor progression, increasing tumor burden in mCRC animal models, suggesting that CCL24 derived from tumor cells promotes tumor progression. H–J) Exogenous addition of circulating rhCCL24 chemokine can promote tumor progression in LOVO mCRC animal experiments, suggesting that CCL24 promotes tumor progression. K–N) Knockdown of HCT116 cell lines with high endogenous expression showed that knockdown of CCL24 in HCT116 cells significantly reduced tumor burden in mCRC animal models and enhanced the therapeutic effect of Bev treatment in mice. ns, not significant; *, *P* < 0.05; **, *P* < 0.01, ***, *P* < 0.001 when compared to the control groups.

To further clarify the source of the CCL24 chemokines, we constructed a BALB/c nude mouse CRC animal model (Figure S2, Supporting Information). Overexpression of CCL24 in LOVO cells resulted in a significant increase in tumor burden and decrease in survival rate of the mCRC animal model, including an increase in tumor size (**Figure** **2**A), liver weight (Figure 2B), and estimated metastasis site (Figure 2B), and decrease in OS (HR = 4.64, P = 0.002, Figure 2C). These results suggested that increased CCL24 expression promotes tumor progression. To rule out interference from other sources of CCL24 and the mouse homolog Ccl24, an adeno‐associated virus vector, AAV8, was constructed to knock out mouse liver Ccl24 and LOVO cells overexpressing CCL24 were implanted. After eliminating the interference of endogenous CCL24 chemokines, overexpression of CCL24 in LOVO cells significantly promoted tumor progression and increased tumor burden in the mCRC animal model. Therefore, tumor cell‐derived CCL24 promotes tumor progression (Figure 2D–G). Addition of cyclic rhCCL24 chemokines promoted tumor progression in LOVO mCRC animal experiments, which further suggested that CCL24 promoted tumor progression (Figure 2H–J). We knocked down the HCT116 cell line with high endogenous expression and the results showed that knocking down CCL24 in HCT116 cells resulted in a significant reduction in the tumor burden of mCRC animal model mice, while enhancing the therapeutic effect of Bev treatment in mice (Figure 2K–N).

### 2.3 Hepatic Stellate Cells (HSCs) Differentiated as iCAFs via CCL24/CCR3/NF‐κB Pathway

**Figure 3:**
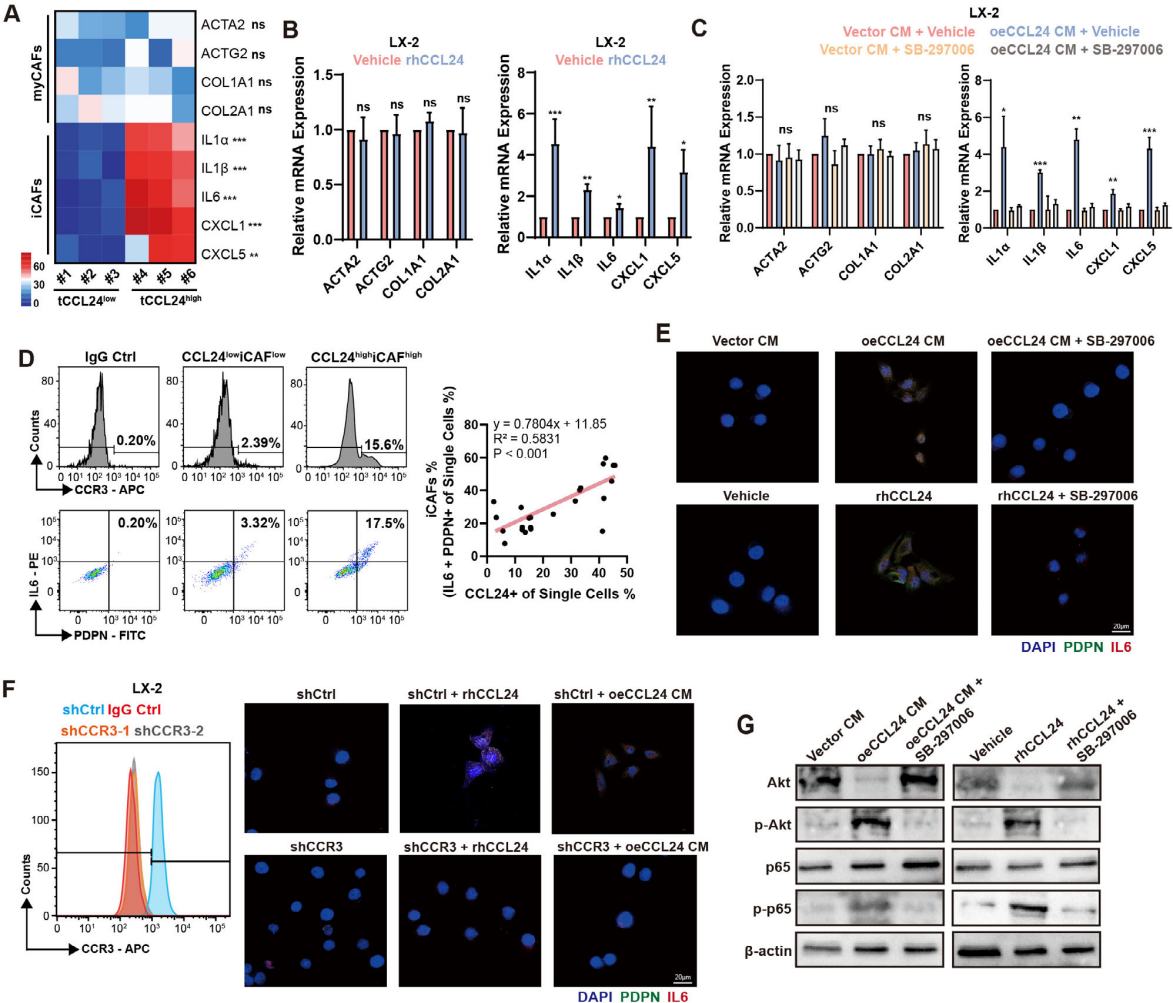
HSCs differentiated as iCAFs via CCL24/CCR3/NF‐κB pathway. A) In mCRC tissues with high expression of CCL24, markers of iCAFs (IL1A, IL1B, IL6, CXCL1, CXCL5) are significantly highly expressed, indicating a high degree of iCAFs infiltration in the TME; The scale bar and the numbers indicates the FPKM of the genes expression. The red color implied the gene was potentially highly expression in the indicated group. B,C) Exogenous addition of rhCCL24 cytokine to the HSC cell line LX‐2 results in significant upregulation of iCAFs markers, while there is no significant change in myCAFs markers; D) CCL24+ cells are positively correlated with the proportion of iCAFs (IL1A+ PDPN+), suggesting that increased expression of CCL24 induces the formation of iCAFs cells in the TME. E) After exogenously adding oeCCL24 LOVO cell culture supernatant or recombinant CCL24 chemokine to the LX‐2 cell culture medium and continuing to culture for 72 h, the expression of PDPN and IL1A in LX‐2 cells is significantly upregulated. F) After knocking out CCR3 in HSC cells, exogenous addition of oeCCL24 LOVO cell culture supernatant or recombinant CCL24 chemokine does not result in high expression of CAF markers in LX‐2 cells, suggesting that CCL24 promotes the differentiation of LX‐2 into iCAFs through the CCR3 receptor. G) Adding oeCCL24 CM in LOVO cell culture supernatant to LX‐2 cells showed that the NF‐κB signaling pathway was activated. ns, not significant; *, *P* < 0.05; **, *P* < 0.01, ***, *P* < 0.001 when compared to the control groups.

Surgical specimens, including liver tumor tissues, from six patients with mCRC were used. Three patients had high expression of CCL24 in their tumors and the remaining three had low expression of CCL24 in their metastatic tumor tissues. Using next‐generation sequencing, we found that in tumor tissues with high CCL24 expression, iCAF markers (IL1A, IL1B, IL6, CXCL1, and CXCL5) were significantly highly expressed, indicating high infiltration of iCAFs in the TME (**Figure** **3**A). Subsequently, we added indigenous rhCCL24 to the HSC cell line LX‐2 and performed PCR analysis. After the addition of rhCCL24 or LOVO^oeCCL24^ culture medium, LX‐2 cells displayed a significant upregulation of iCAFs markers, whereas there was no significant change in myCAF markers. These results suggested that upon stimulation with CCL24, HSCs differentiated into the iCAFs cell subtype (Figure 3B,C). We collected fresh mCRC tissues and performed flow cytometry analysis and found that CCL24+ cells were positively correlated with the proportion of iCAFs (IL1A+PDPN+) (P<0.001), suggesting that increased CCL24 expression induced the formation of TME iCAF cells (Figure 3D). After adding the homogenous oeCCL24 LOVO cell culture supernatant or recombinant CCL24 chemokine to the LX‐2 cell culture medium and culturing for 72 h, the expression of PDPN and IL1A in LX‐2 cells was significantly upregulated (Figure 3E). To demonstrate that CCL24 signals through the HSC cell surface CCR3 receptor, we used shRNA to knock out CCR3 on the LX‐2 cell membrane. After knocking out CCR3, adding Exogenous oeCCL24 to LOVO cell culture supernatant or recombinant CCL24 chemokine did not result in high expression of CAF markers in LX‐2 cells, suggesting that CCL24 promotes differentiation of LX‐2 into iCAFs through the CCR3 receptor (Figure 3F). Adding exogenous oeCCL24 CM in LOVO cell culture supernatant to LX‐2 cells showed that the NF‐κB signaling pathway was activated and using the CCR3 receptor blocker, SB‐297006, antagonized the activation of the NF‐κB signaling pathway induced by CCL24 (Figure 3G).

The tube formation assay on HUVECs showed that tumor cells containing CCL24 promoted vessel formation (Figure S3A, Supporting Information). In the TME, with a higher proportion of iCAF infiltration, elevated concentrations of IL‐1α and IL‐6 chemokines were detected (Figure S3B, Supporting Information). The combined use of IL‐1R and CCR3 inhibitors significantly suppressed tumor progression in liver metastasis (Figure S3C, Supporting Information), liver weight (Figure S3D, Supporting Information), and the estimated metastasis site (Figure S3E, Supporting Information), and improved OS (Figure S3F, Supporting Information). A representative hematoxylin‐eosin (HE) staining image of liver metastasis is shown in Figure S3G (Supporting Information).

### 2.4 Construction of the Patient‐Derived Bioengineered Extraceullar Vehicles and Delivering the siRNAs to the Tumor Tissue

**Figure 4:**
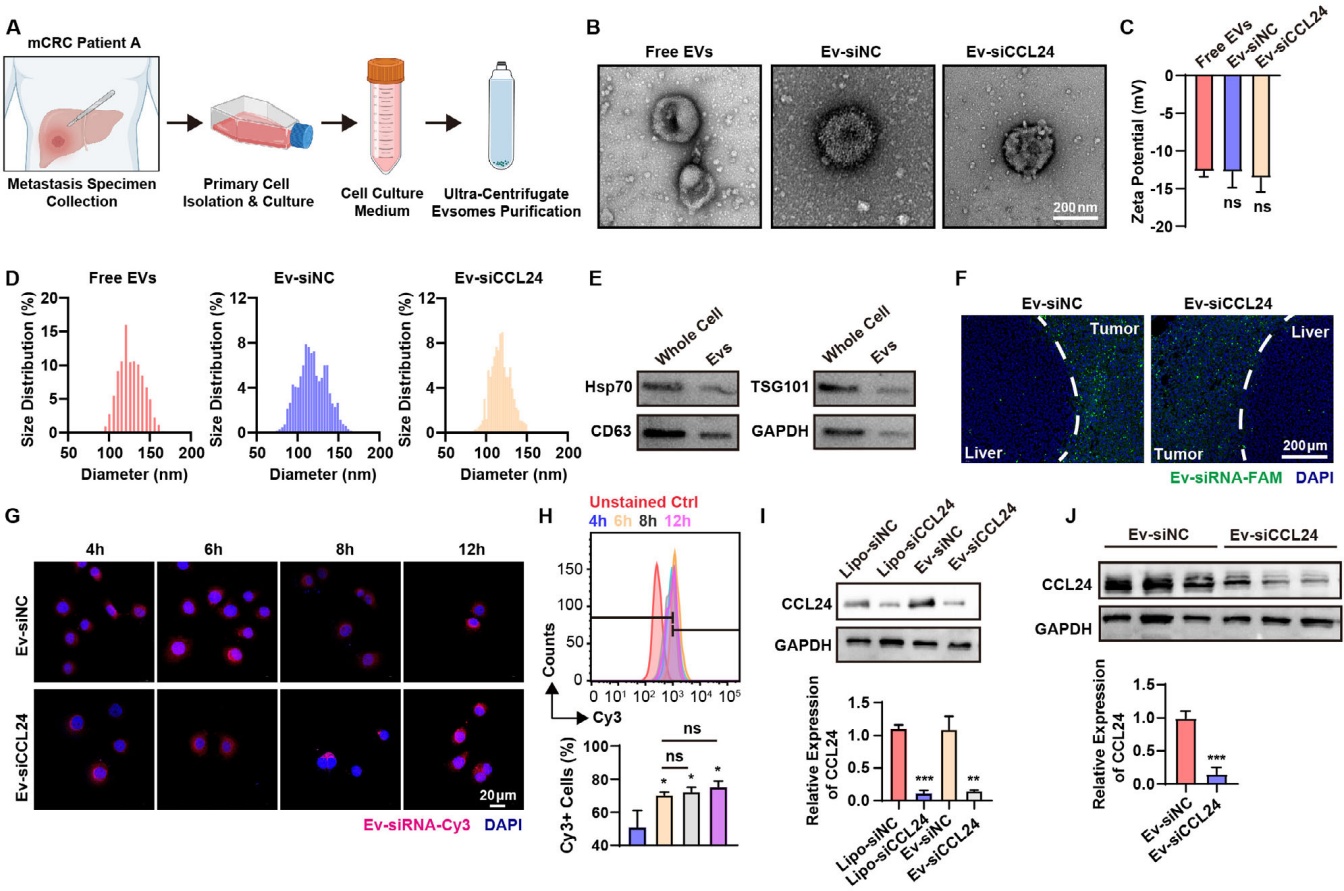
Construction of the patient‐derived bioengineered extraceullar vehicles (EVs) and delivering the siRNAs to the tumor tissue. A) Schematic illustration of preparing primary cell culture and purifying EVs from primary cells. B) The representative TEM images of the EVs and EVs loading siRNAs. C) The zeta potential of the bioengineered EVs. D) The size distribution of the EVs. E) The western‐blot analysis was performed to verify the expression of the EV markers. F) The in vivo distribution of the bioengineered EVs in PDX mice model constructed from the same patients. The cellular uptake assays were performed by confocal microscopy (G) and flow cytometry analysis (H). Western blotting (I) and qPCR (J) assay indicated that the gene knockdown ability of the bioengineered EVs in vitro. ns, not significant; *, *P* < 0.05; **, *P* < 0.01, ***, *P* < 0.001 when compared to the control groups.

Extracellular vesicles (EVs), which transport non‐coding RNAs or proteins, serve as an important communication medium between cancer cells. To facilitate the delivery of siRNAs with superior biocompatibility and enhanced safety compared to alternative chemical agents, extracellular vesicles were isolated and purified from metastatic liver tumor cells derived from patients with CRC (**Figure** **4**A). Extracellular vesicles were transfected with *CCL24* siRNA or a negative control (siNC). The extracellular vesicles were photographed and measured using transmission electron microscopy (Figure 4B). The zeta potential and size distribution were measured using nanoparticle tracking analysis system (Figure 4C,D). Western blotting was performed to verify that the expression of EVs markers was slightly higher than that in the donor whole cells. However, no significant differences were observed (Figure 4E).

To further study the cellular uptake and biodistribution of the engineered extracellular vesicles, siRNA‐FAM was transfected into the extracellular vesicles. A patient‐derived xenograft (PDX) liver metastasis mouse model was constructed using NOG mice. A mouse model of liver metastasis was established as described previously.^[^ ^20^ ^]^ The in vivo distribution indicated that after intravenous injection of the extracellular vesicle‐siRNA (Ev‐siCCL24 and Ev‐siNC), fluorescence was captured in the mice and siNC was detected at the liver metastasis tumor site 24 h after injection (Figure 4F). Cellular uptake of bioengineered extracellular vesicles was performed and measured using confocal microscopy and flow cytometry. Fluorescence was observed in the cytoplasm of recipient cells. The results indicated that the CRC cell lines and patient's primary cancer cell lines could take up EVs within 4 h (Figure 4G,H). To transport siCCL24, engineered Ev‐siCCL24 was added to cell culture plates. Western blotting and RT‐qPCR assays demonstrated that the expression of CCL24 was significantly downregulated in the primary cells of patients with CRC (Figure 4I,J).

To determine the loading efficiency of siRNA in extracellular vesicles, siRNAs were labeled with Cy5 fluorescence, which was evaluated using a microplate reader and calculated based on a precalculated standard curve (Figure S4, Supporting Information). The loading capacity of siRNAs in 10^10^ extracellular vesicles was 0.320 ± 0.010 µg. Collectively, these results implied that the extracellular vesicles from patients with cancer could effectively load siRNAs without changing the structure and physicochemical properties of the extracellular vesicles to obtain Ev‐siRNA nanoformulations. To determine the distribution of the bio‐engineered EVs, we injected EV‐siNC‐Cy5 or free siNC‐Cy5 into the PDX mice. The fluorescence imaging showed accumulation in the tumor with the EVs transporter. Without the EVs, the no fluorescence was detected in the major organs (Figure S5, Supporting Information). The results implied that the EVs may have ideal tumor‐specific acclamation accounting for the specific feature of tumor‐derived EVs.

### 2.5 The Antitumor Activity of Ev‐siRNA in CRC Antiangiogenic Therapy Resistance in PDX Mouse Models

**Figure 5:**
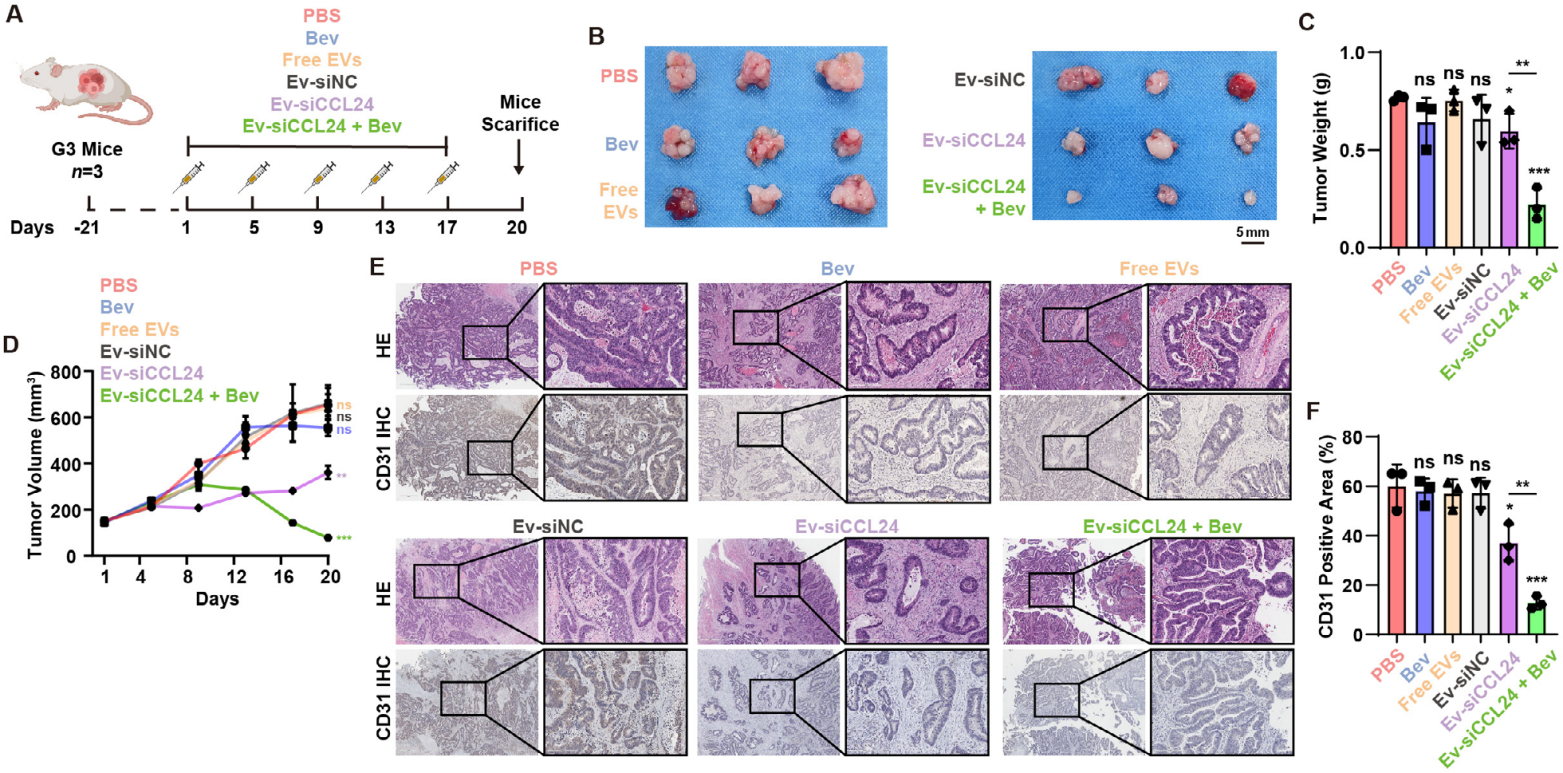
The anti‐tumor activity of Ev‐siRNA in CRC antiangiogenic therapy resistance in PDX mouse models. A) Schematic illustration of establishing PDX model and illustration of treatment schedule. B) The images of the resected tumors in the mice. C) The tumor weight of the mice at the endpoint of the in vivo experiments, and tumor inhibition profiles of the mice receiving different treatments. Mean ± SD (*n* = 3). D) The tumor volume of the mice in each group. E) The representative HE and CD31 IHC staining images among the groups. F) The estimated CD31 positive area in IHC imagews. Scale bar = 600 µm for 40× magnification images and 200 µm for 200× magnification images. ns, not significant; *, *P* < 0.05; **, *P* < 0.01, ***, *P* < 0.001 when compared to the control groups.

To evaluate the antitumor activity of Ev‐siCCL24 in a chemoresistant mouse model, we generated a PDX mouse model. Surgical specimens from a chemoresistant patients with CRC recurrence were collected and implanted into NOG mice for PDX construction. Tumors from the mice were resected and passed through three generations. The LX‐2 cells were subcutaneously injected. Primary cells from the same patient were collected and cultured for EV enrichment (**Figure** **5**A). The mice received the therapy; the treatment protocol is shown in Figure 5A.

As shown in Figure 5B,C, treatment with Bev alone did not achieve a significant therapeutic effect in the mCRC PDX model. However, the combination of Ev‐siCCL24 and Bev resulted in significant tumor shrinkage in the PDX mouse model. Notably, the single use of free extracellular vesicles or Ev‐siCCL24 may not acquire a therapeutic effect in the PDX mouse model. These results imply that the knockdown of CCL24 may not directly induce cell apoptosis but may enhance the therapeutic effect of antiangiogenic therapy reagents. All mice survived until the end of the experiments, and their tumors were harvested on day 20 after the first treatment and stained for HE and CD31 (IHC analysis). The results indicated that CD31 was highly expressed in the control group, and tumor proliferation was significantly inhibited by the combination treatment of Ev‐siCCL24 and Bev (Figure 5F), indicating that the combination of CCL24 knockdown and anti‐angiogenic therapy may hinder the formation of tumor‐related neovasculature. Collectively, our Evsome delivery system could effectively deliver siRNAs to the tumor site in a more clinically relevant PDX model, and the delivery of siCCL24 via extracellular vesicles may significantly enhance sensitivity to antiangiogenic reagents. All the mice survived until the end of the experiment. Major organs and blood samples were collected. Biosafety analysis indicated the EVs did not cause hemolytic reaction (Figure S6, Supporting Information) and displayedno external toxicity to the major organs (Figure S7, Supporting Information).

### 2.6 In Vivo Therapeutic Efficacy of Ev‐siCCL24 Against Colorectal Cancer Liver Metastasis

**Figure 6:**
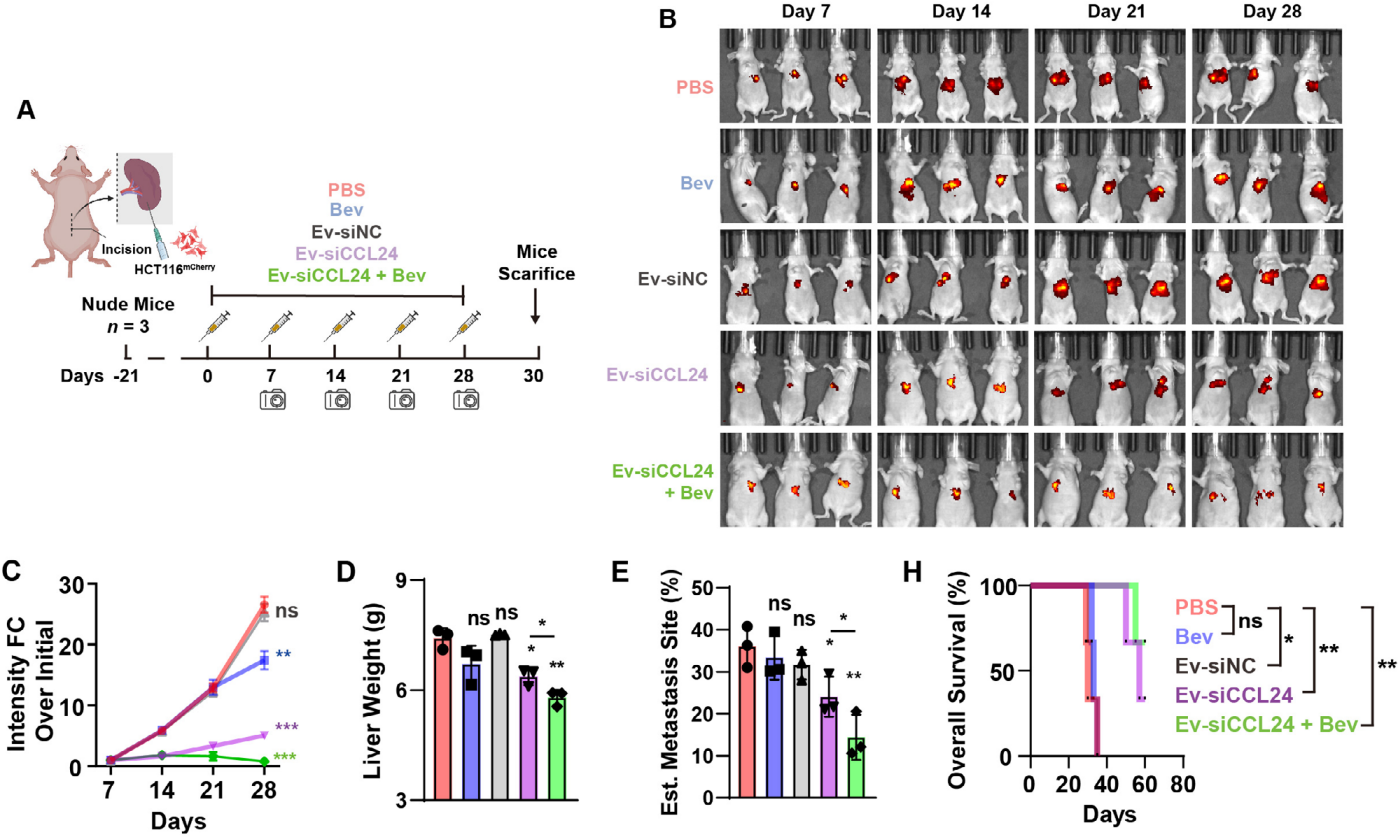
In vivo therapeutic efficacy of Exo‐siCCL24 against CRC liver metastasis. A) Schematic illustration of constructing CRC liver metastasis mice model by using the HCT116R‐mCherry cell lines and purifying EVs and the treatment protocol among the groups. B) The in vivo fluorescence of HCT116^R^ tumor‐bearing mice at different time pointes. C) The measurement of the in vivo fluorescence intensity of HCT116^R^ tumor‐bearing mice at different time pointes. D) The liver weight in each group at the end of the in vivo experiments. E) The estimated metastasis site in each group at the end of the in vivo experiments. H) The survival analysis of the in vivo experiments. ns, not significant; *, *P* < 0.05; **, *P* < 0.01, ***, *P* < 0.001 when compared to the control groups.

To further evaluate the effects of CRCLM suppression, a mouse model of liver metastasis was established using female nude mice. HCT116^R^ cells were transfected with a lentivirus carrying mCherry. Stable expression of the mCherry HCT116^R^ cell lines (HCT116^R^ – mCherry) was achieved and injected into the spleen to construct a mouse liver metastasis mouse model. Various formulations were administered to the mice (**Figure** **6**A). To further observe liver metastasis, the tumor was observed and measured using in vivo imaging of the mCherry fluorescence signal intensity. As shown in Figure 6B–E, the combined use of Ev‐siCCL24 and Bev significantly suppressed liver metastasis in the mouse model. The mice were administered the formulations and survival analyses were conducted. The mice were sacrificed 80 days after the first treatment. The livers were carefully resected at the end of the experiment or from dead mice. A representative image of the resected liver and its corresponding HE and IF staining of the CD31 marker are shown in Figure S8 (Supporting Information). The CD31 staining analysis revealed that the combination usage of Ev‐siCCL24 and Bev siginificantly reduced the vessel formation in metastasic tumor tissue. The amount and size of the metastatic tumors in the liver were significantly larger in the control group after only administering PBS solution, and OXA may not have a significant impact on liver metastasis and chemoresistance in a mouse model. The knockdown of CCL24 may significantly increase the therapeutic efficacy of Bev when combining Ev‐siCCL24 and Bev. These results imply that the combination of CCL24 knockdown and anti‐angiogenic therapy may hinder the formation of tumor‐related vessels. In the survival analysis, the prognosis of the mice that received the combination was significantly better than that of the control group (Figure 6H).

## 3 Discussion and Conclusion

The combination of chemotherapy and anti‐VEGF antibodies is considered the critical standard treatment for patients with mCRC. However, the overall therapeutic effect in patients with CRC may be limited due to the development of therapy resistance. The TME plays an important role in tumor progression and distant metastasis. Thus, identifying the key molecular driver events associated with tumor therapy resistance may allow for innovative therapeutic strategies that may substantially contribute to the prognosis of patients with metastatic CRC. In the present study, we used RNA sequencing to identify key genes associated with potential Bev therapy in a cohort of patients with mCRC. We demonstrated that CCL24 chemokines were overexpressed in metastatic and therapy‐resistant clinical specimens and validated this in a larger cohort of patients with CRC. CCL24 regulates the CCR3/NK‐κB molecular pathway to promote iCAF formation, a subpopulation of CAFs that promotes the formation of tumor‐related vessels and induces resistance to antiangiogenic therapy. Based on these results, we designed a metastatic CRC primary cell‐derived extracellular vesicle delivery system for the simultaneous delivery of siRNAs targeting CCL24 into the TME. The extracellular vesicle delivery system displayed ideal therapeutic effects in a therapy‐resistant PDX model. Tumor‐bearing mice treated with bioengineered extracellular vesicles and subjected to hepatectomy showed ideal OS.

The role of the eotaxin family members in cancer progression and distant metastasis remains controversial. CCL24 is upregulated in allergic and inflammatory diseases.^[^ ^21^ ^]^ However, the role of CCL24 in cancer has not yet been fully demonstrated. In the current study, we found a high expression of CCL24 in tumor and distant metastatic tumor tissues, which may contribute to drug resistance and tumor immune evasion. A high concentration of CCL24 in the serum may act as a biomarker for tumor recurrence in CRC. Our study demonstrated that higher CCL24 expression may contribute to therapeutic resistance and distant liver metastasis. CCL24 inhibition causes significant therapy resistance in CRC cells in vivo. CCL24 inhibition exhibits a synergistic effect with chemotherapy drugs, suggesting the potential therapeutic value of CCL24 for anti‐angiogenic therapy and liver metastasis in CRC.

Delivering siRNA is a promising way to target liver metastasis and/or chemoresistance in CRC.^[^ ^17b^ ^]^ Based on the current study, we suggest that supplementation with Ev‐siCCL24 can effectively prevent metastatic tumor recurrence in a PDX mouse model. These results provide new insights and innovations in clinical practice for targeting chemoresistant and metastatic tumors. Although we suggested an innovative siRNA therapeutic method for overcoming chemoresistance in metastatic CRC, there are some limitations. CCL24 may have different crossroads in its molecular pathway. Therefore, the underlying molecular mechanisms should be further studied. More importantly, the findings of the current study were mainly based on PDX models. However, these results have not been validated in clinical practice. Therefore, further translational studies are required.

In conclusion, we enrolled patients with recurrent CRC with liver metastasis and collected clinical specimens for next‐generation RNA sequencing to identify potential therapeutic targets for distant metastasis and resistance to antiangiogenic therapy. CCL24 was found to be highly related to CRC liver metastasis and chemoresistance, which was further validated via bioinformatics analysis and clinical specimens. Slicing CCL24 significantly increased the sensitivity of the mouse model of liver metastasis to anti‐angiogenic therapeutic agents. We designed a primary cell‐derived extracellular vesicle delivery system for the simultaneous delivery of siRNAs targeting CCL24 and increased sensitivity to chemotherapy reagents. The engineered extracellular vesicles exerted an ideal antitumor effect in CRCLM and PDX mouse models. Our results identified a novel therapeutic target for distant metastasis in patients with CRC and suggested a possible therapeutic alternative for patients with CRC with therapy resistance and distant metastasis.

## 4 Experimental Section

### Clinical Specimens Collection

This study enrolled 42 CRC patients surgical tumor specimen, with an additional four CRC patients included in the PDX model construction. Primary cells from CRC patients were extracted from tumor tissue for subsequent experiments. Additional blood sample from another CRC patient cohort were also collected. The tumor and blood samples were acquired during palliative or radical surgical procedures at the Guangdong Provincial People's Hospital (Guangdong Academy of Medical Sciences). All participants signed written consent forms prior to specimen collection. The inclusion criteria for CRC patients were as follows: 1) patients who underwent palliative or radical surgery, with pathological confirmation of colorectal adenocarcinoma. 2) Fresh tumor tissue was harvested directly from the tumor site. Tumor staging was performed according to the 8th edition of the TNM staging system. To evaluate treatment efficacy, radiological examinations were conducted using the following criteria: partial response (PR), complete response (CR), stable disease (SD), and progressive disease (PD). For this study, PR and CR were classified as therapy responses, while SD and PD were assigned as therapy‐resistant.

### RNA‐seq Analysis

To identify the potential key regulators of CRCLM, tumor samples from chemotherapy‐resistant CRC patients with liver metastases were collected, including their primary CRC tissues and recurrent CRCLM tissues. RNA sequence analysis (RNA‐seq) was then performed. Clinical samples were excised from the surgery and stored in an RNA store reagent (Beyotime, Shanghai, China). Total RNA was isolated from the tissue samples using TRIzol reagent (Invitrogen, USA) according to the manufacturer's protocol. RNA integrity and DNA contamination were assessed by agarose gel electrophoresis. The TruSeq Stranded Total RNA Library Prep Kit (Illumina, San Diego, CA, USA) was used to construct RNA‐seq libraries according to the manufacturer's instructions. Sequencing was conducted using Illumina HiSeq 2000.

### In Vivo Therapeutic Efficiency and Construction of CRC Liver Metastasis and PDX Model

To construct a PDX mouse model, tumor samples from CRC patients were obtained through surgery at Guangdong Provincial People's Hospital. The samples were thawed and cut into 3 mm × 3 mm × 3 mm pieces. These pieces were then inserted under the skin of the NOG mice, with each mouse receiving one or two pieces. When the tumors grew to 1200 mm^3^, they were extracted to expand the cohort. The tumors were then divided and transplanted beneath the skin of the final group of mice (five per group), which were then administered the appropriate treatments. After euthanizing the mice, a histological examination was performed. All operations were conducted under general anesthesia to construct a liver metastasis mouse model. A 2‐cm incision along the midline to enter the abdominal cavity, and a retractor was placed. The spleen was exposed on gauze, and 5 × 10^4^ HCT116R – mCherry cells in 250 µL of PRIM‐1640 medium were injected into the center of the spleen for 15 s. The needle remained in place for 2 min after injection. The spleen was then removed and the abdominal wall was closed by stitching the muscle layer followed by the skin.

### Statistical Analysis

The results are expressed as mean ± standard deviation (SD). Student's *t*‐test or unpaired t‐test was employed to compare two groups. For comparisons involving three or more groups, one‐way ANOVA was used. The relationship between CCL24 levels and clinicopathological characteristics was evaluated using the chi‐squared (χ2) test. Kaplan‐Meier analysis and log‐rank tests were used to assess survival. Hazard ratios (HR) and 95% confidence intervals (CI) were calculated to evaluate patient survival. Cox univariate and multivariate analyses were conducted to identify the potential survival variables. Statistical analyses were performed using GraphPad Prism 9.3 (GraphPad Software, La Jolla, CA, USA). Statistical significance was set at *P* < 0.05. In the presented results, “ns” indicates no statistical significance, “*” denotes *P*<0.05, “**” represents *P*<0.01, and “***” indicates *P*<0.001 when compared to the control group or the wild‐type (WT) group.

### Ethical Approval

The study protocol for collecting clinical specimens was approved by the Institutional Ethics Committee of Guangdong Provincial People's Hospital (Guangdong Academy of Medical Sciences) under the grant number of GDREC2020061H. The in vivo study was approved by the Ethics Committee of Guangdong Provincial People's Hospital (Guangdong Academy of Medical Sciences) under the grant number of KY2024‐892‐01.

## Conflict of Interest

The authors declare no conflict of interest.

## Author Contributions

J.W., C.L., P.W., Z.L., and Z.L. carried out the laboratory research, methodology, formal analysis, and writing the primary version of manuscript; J.W., C.L., P.W., Z.L., and C.H. contributed to validation and data curation; Z.L., C.H., X.Y. contributed to image editing; and C.H., X.Y. contributed to projection design, conceptualization, and supervision.

## Supporting information



Supporting Information

## Data Availability

The data that support the findings of this study are available on request from the corresponding author. The data are not publicly available due to privacy or ethical restrictions.
