# Supplementary material for: Bioengineered Tumor‐Derived Extracellular Vehicles Suppressed Colorectal Cancer Liver Metastasis and Bevacizumab Resistance
Source: Adv Sci (Weinh). 2025 May 21;12(23):2417714. doi: 10.1002/advs.202417714 (PMC12199312; doi:10.1002/advs.202417714)
Supplement: Supplementary file 1 — Supporting Information [file ADVS-12-2417714-s001.pdf]

ADVANCED SCIENCE
Open Access

# Supporting Information

for *Adv. Sci.*, DOI 10.1002/advs.202417714

Bioengineered Tumor-Derived Extracellular Vehicles Suppressed Colorectal Cancer Liver Metastasis and Bevacizumab Resistance

*Junjiang Wang, Chunsheng Liu, Ping Wang, Zhiyuan Liu, Weixian Hu, Zejian Lv*, Chengzhi Huang* and Xueqing Yao**

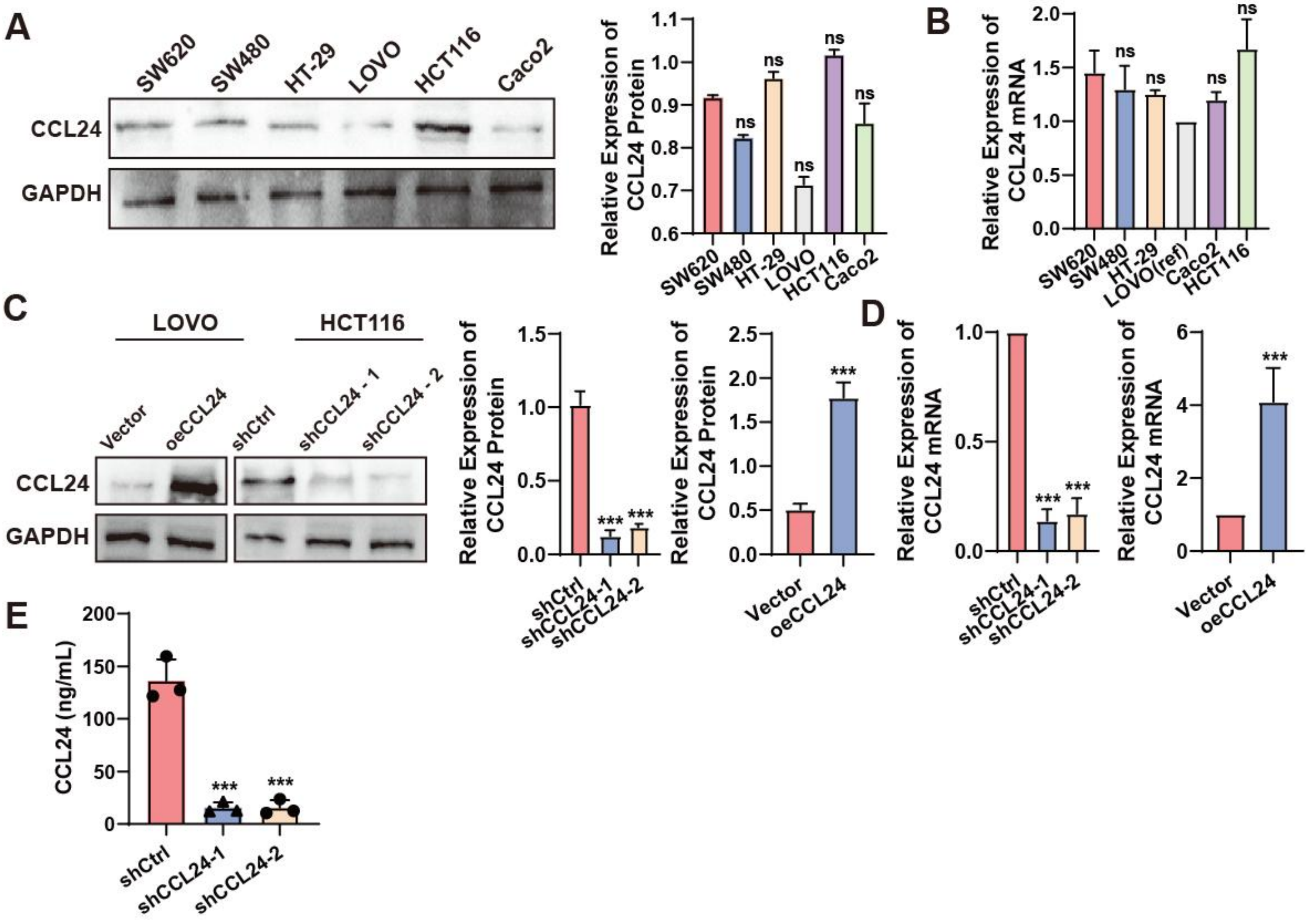

**Figure S1 | Knockdown of CCL24 in tumor cell lines.**

The expression of CCL24 chemokines in CRC cell lines detected by Western-blotting (A) and PCR (B). The HCT116 cell line exhibited a higher level of CCL24 expression, while LOVO demostrated lower expression of CCL24. (C) The stable knockdown of CCL24 by siRNAs and plasmids. The expression of CCL24 was detected by Western-blotting and PCR analysis. (E) The supernatant of HCT116 culture medium indicated that the CCL24 significantly decreased after the slicing CCL24 mRNA. ns, not significant; ***, P<0.001 in the statistics analysis when compared to the control groups.

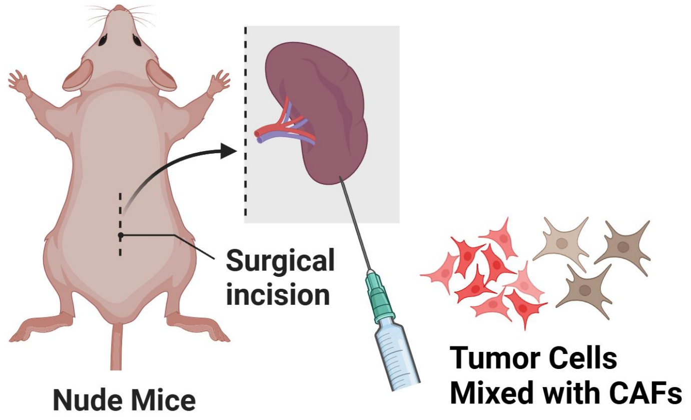


**Figure S2 | The construction model of liver metastasis CRC mice model.**

The human tumor cell lines and human CAF cells were injected into spleen to construct the CRC liver metastasis mice model.

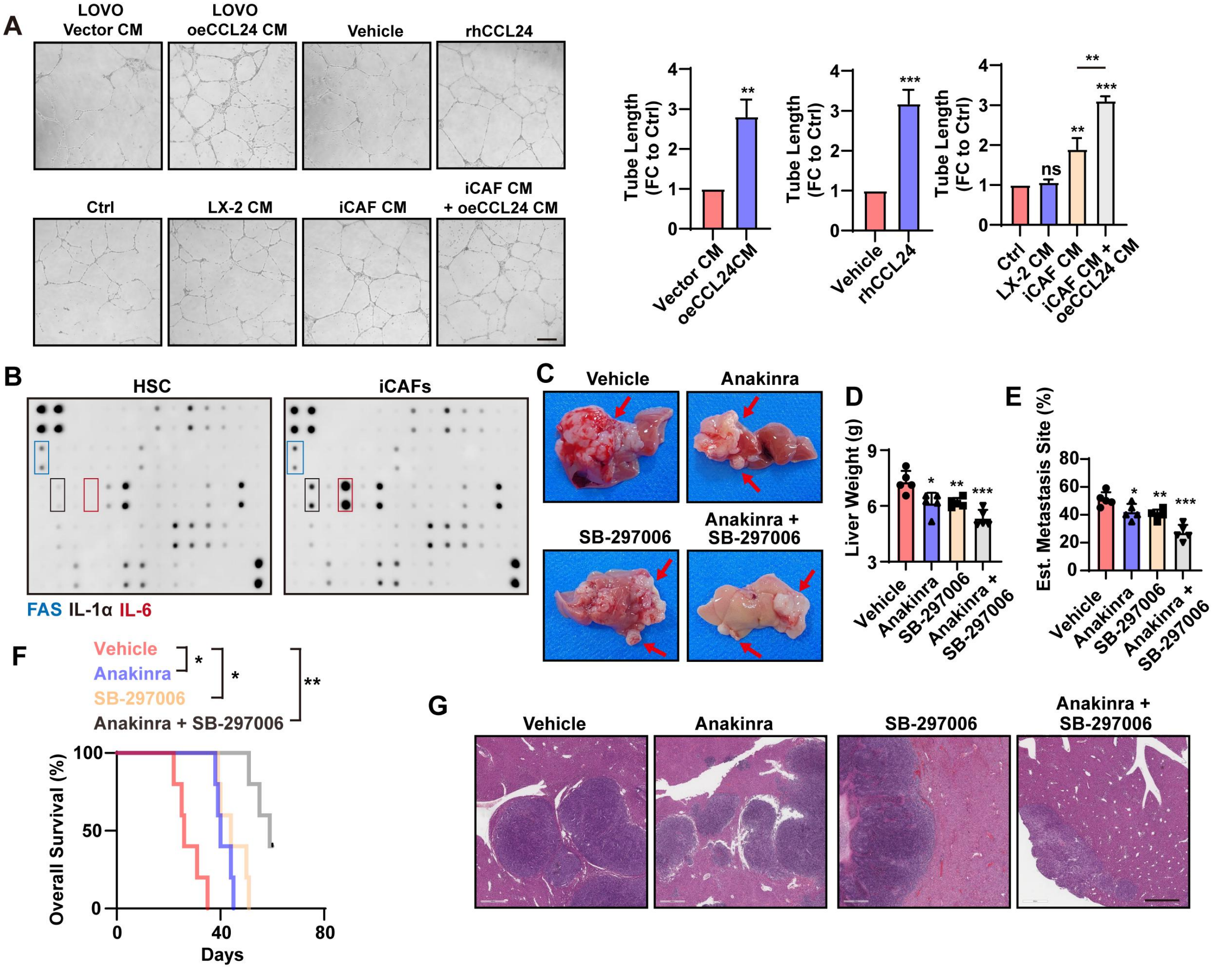


**Figure S3 | HSCs differentiated as iCAFs via CCL24/CCR3/NF-κB pathway.**

(A) Exogenous addition of oeCCL24 LOVO cell culture supernatant to LX-2 cells shows activation of the NF-κB signaling pathway, and the CCR3 receptor blocker SB-297006 can antagonize the activation of the NF-κB signaling pathway induced by CCL24. The tube formation assay implied that the tumor cell orginated CCL24 promote vessle formation. (B) In the tumor microenvironment with elevated iCAFs proportion, the levels of cytokines such as IL-1α and IL-6 are significantly increased. IL-1α and tumor-derived CCL24 bind to IL-1R and CCR3 receptors on the surface of HUVEC cells, respectively, promoting tumor-associated angiogenesis and antagonizing the therapeutic effect of Bev, mediating resistance to Bev treatment; The in vivo experiments suggest that the use of IL-1R and CCR3 receptor inhibitors significantly improves the therapeutic effect of Bev in mCRC. (C) The representive image, the weight (D), estimated metastasis site of resected liver (E). (F) The combination of SB-297006 and Anakinra significantly improve the therapy effect in mCRC mice model. (G) The representative HE staining of the resected metastasic tumor tissue, Scale bar = 500 μm. ns, not significant; *, P<0.05; **, P<0.01, ***, P<0.001 when compared to the control groups.

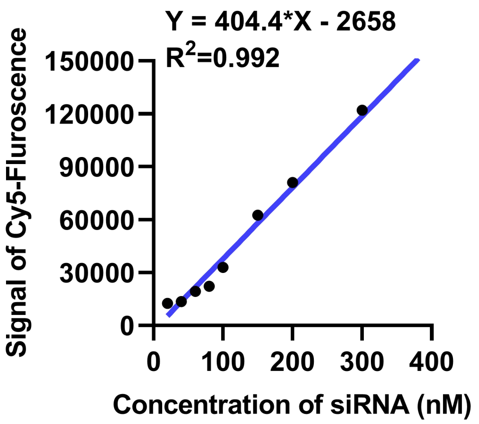


**Figure S4 | The standard curves of siRNA concentration and signal intensity of Cy5 fluorescence.**

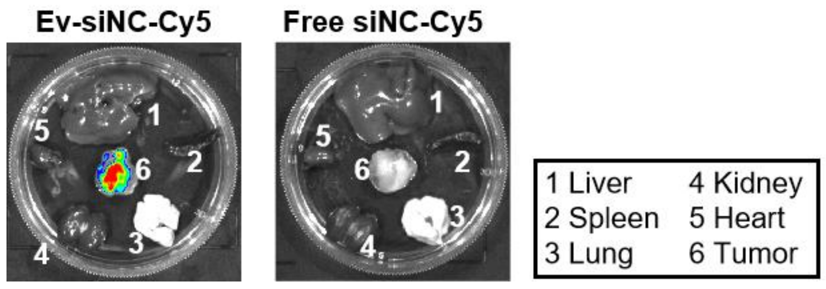


**Figure S5 | The biodistribution analysis of the bioengineered EVs in PDX model.**

At 4 h after i.v. injection of EV-siNC-Cy5 or free siNC-cy5, fluorescence imaging showed accumulation in the tumor with the EVs transporter. Without the EVs, the no fluorescence was detected in the major organs.

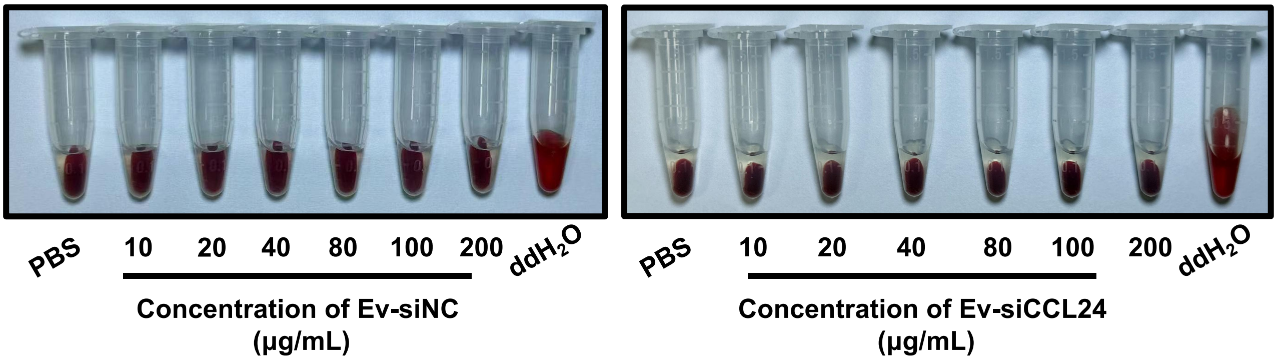


**Figure S6 | The bio-compatibility test of bioengineered EVs in blood circulation.**

The bioengineered EVs populations did not cause hemolytic reaction *in vitro*.

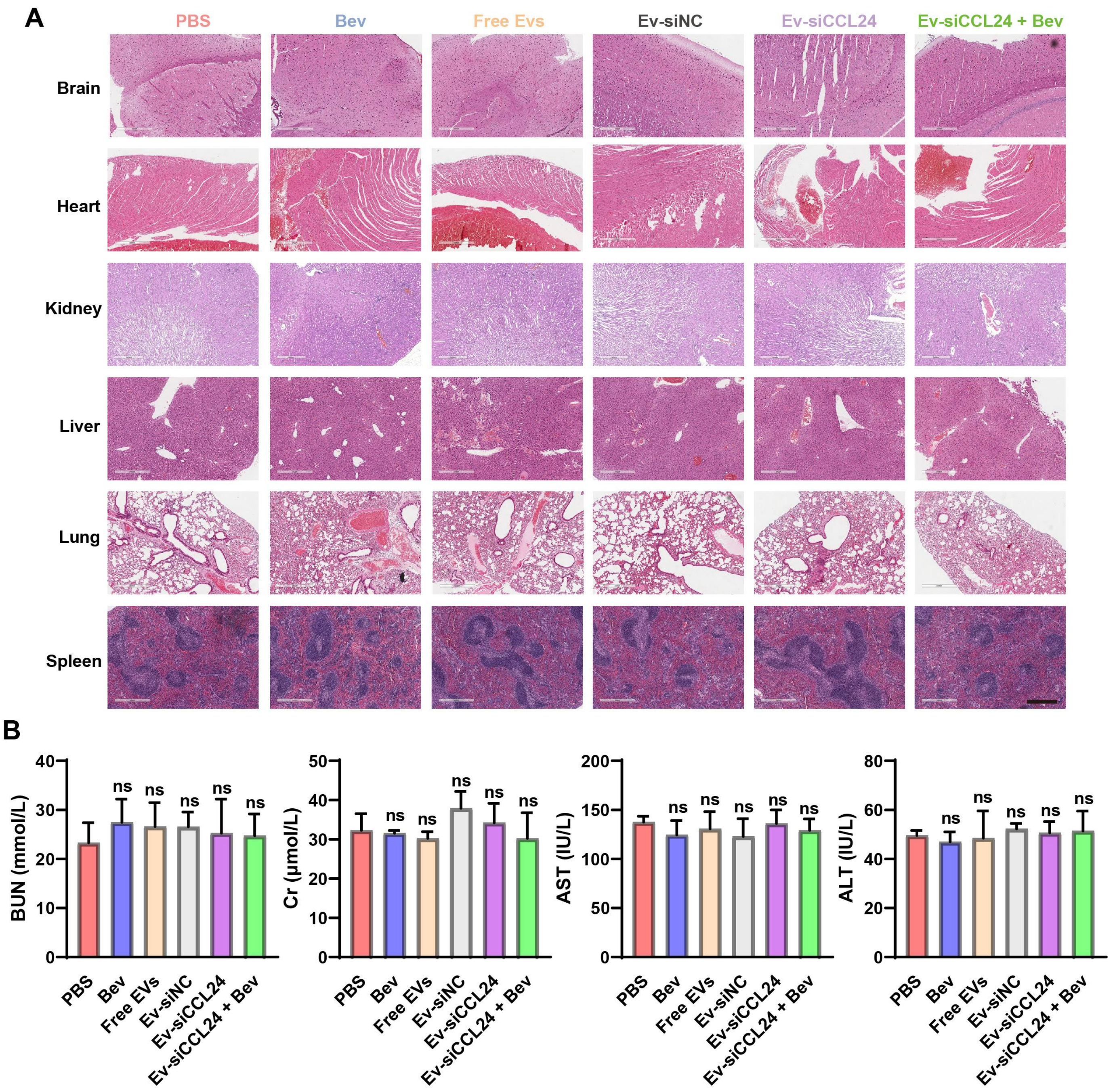


**Figure S7 | Biosafety analysis of the bioengineered EVs in PDX model.**

(A) The HE staining analysis revealed that no obvious toxicity was found in the major organs and all the PDX mice survived at the end of the *in vivo* experiments, Scale bar = 600 μm. (B) The serum tests of BUN, Cr, AST, ALT revealed that there were no obvious changes among the groups. ns, not significant

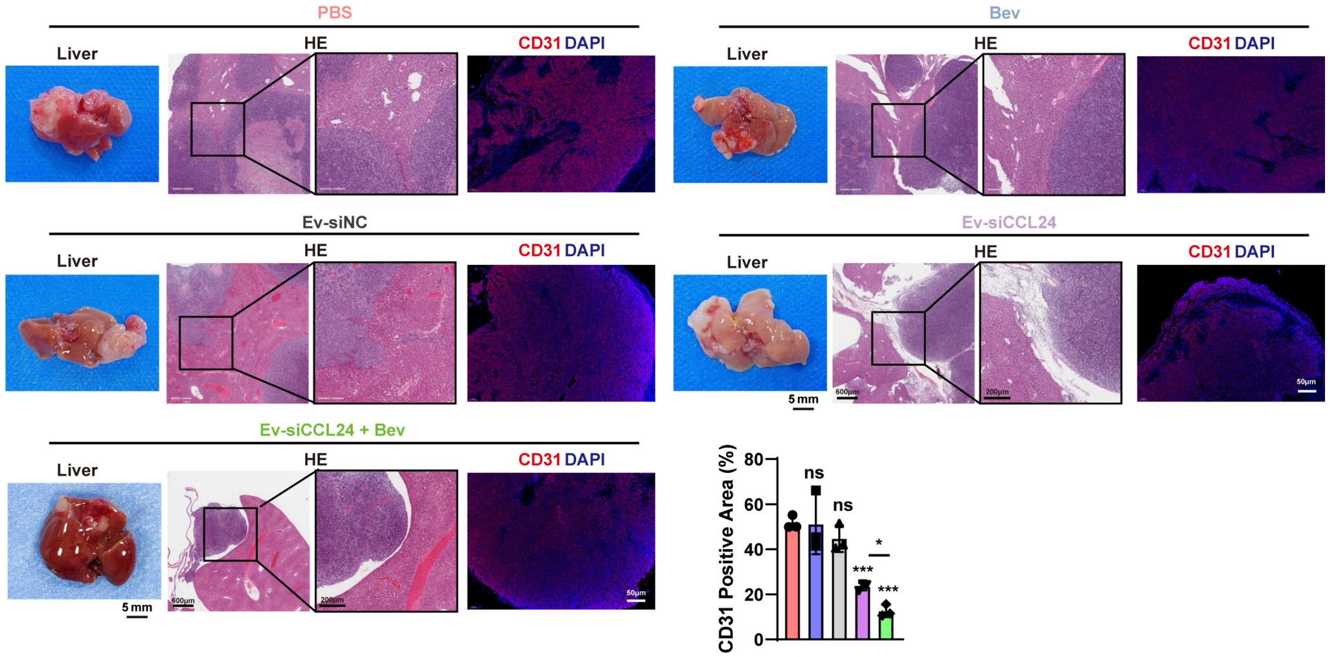


**Figure S8 | The images of the resected liver metastatic tumors and its corresponding HE staining, IF analysis .**

The HE staining and CD31 IF analysis revealed that the combination usage of Ev-siCCL24 and Bev siginificantly reduced the vessel formation in metastasic tumor tissue. The scale bar have been marked in the images. ns, not significant; *, P<0.05; ***, P<0.001 when compared to the control groups.

**Table S1 |** Clinical Information on patients whose tumors were collected for RNA sequence.

| Patient No. | Age | Gender | Primary Tumor Location | Pathology Type | Date of Primary Tumor Specimen Collection | Date of Metastasis Tumor Specimen Collection |
|---|---|---|---|---|---|---|
| #1 | 45 | Male | Colon | Adenocarcinoma | 2019.3 | 2021.1 |
| #2 | 59 | Female | Rectum | Adenocarcinoma | 2019.5 | 2020.12 |
| #3 | 53 | Male | Colon | Adenocarcinoma | 2020.1 | 2021.5 |
| #4 | 52 | Male | Colon | Adenocarcinoma | 2021.5 | NA |
| #5 | 66 | Female | Colon | Adenocarcinoma | 2021.6 | NA |
| #6 | 71 | Female | Colon | Adenocarcinoma | 2021.5 | NA |

NA: Not available.

**Table S2 |** Sequence of siRNAs sequences used in the research.

| Gene | Sequence |
|---|---|
| siCCL24-1 | Sense: 5′-AAUUCUCUUGGAAACAAAGAA-3′ |
| | Anti-Sense: 5′-CUUUGUUUCCAAGAGAAUUCC-3′ |
| siCCL24-2 | Sense: 5′-AAGAAACAGGAAAAUUAGCUC-3′ |
| | Anti-Sense: 5′-GCUAAUUUUCCUGUUUCUUAG-3′ |
| siNC | Sense: 5′-UUCUUCGAACGUGUCACGUTT-3 |
| | Anti-Sense: 5′-ACGUGACACGUUCGGAGAATT-3′ |

**Table S3 |** Sequence of qRT-PCR Primers used in the research.

| Gene | Primers | Sequence (5'-3') |
|---|---|---|
| CCL24 | Forward | CTGTTACCTCCGGGTCCTTT |
| | Reverse | GGGGAAGCCTTCTTCTGCTT |
| ACTA2 | Forward | GTTCCGCTCCTCTCTCCAAC |
| | Reverse | TAGTCCCGGGGATAGGCAAA |
| ACTG2 | Forward | CGCCCTCGCCACCAGAT |
| | Reverse | CTGTCAGCAATGCCAGGGTA |
| COL1A1 | Forward | TGACGAGACCAAGAACTGCC |
| | Reverse | GCACCATCATTTCCACGAGC |
| COL2A1 | Forward | GTAGAGACCCGGACCCGC |
| | Reverse | ACTCTCCGAAGGGGATCTCA |
| IL1A (IL1α) | Forward | TGGCGTTTGAGTCAGCAAAG |
| | Reverse | AGCACACCCAGTAGTCTTGC |
| IL1B (IL1β) | Forward | TCGCCAGTGAAATGATGGCT |
| | Reverse | TATCCTGTCCCTGGAGGTGG |
| IL6 | Forward | CCTTCGGTCCAGTTGCCTT |
| | Reverse | AGCTGCGCAGAATGAGATGA |
| CXCL1 | Forward | CTGGCGGATCCAAGCAAATG |
| | Reverse | GCCCCTTTGTTCTAAGCCAG |
| CXCL5 | Forward | AAGTTCCCTCCCCACTCACA |
| | Reverse | GGCATCTAAAAAGCTCAGCAAT |
| GAPDH | Forward | TTCCAGTGGCTGCTGTTGTT |
| | Reverse | TTTCCGGGCGTAAAAGCACT |

**Table S4 |** Antibodies and its dilutions.

| Antigen | Host or Isotype | Manufacturer and Cat. No. | Dilution (x-fold) for Western-Blots | Dilution (x-fold) for IHC or IF | Dilution (x-fold) for Flow Cytometer |
|---|---|---|---|---|---|
| CD31 | Rabbit | Proteintech; 11265-1-AP | Not available | 200 | Not available |
| CCL24 | Rabbit | Proteintech; 22306-1-AP | 1000 | 200 | Not available |
| GAPDH | Rabbit | Abcam; ab9485 | 2000 | Not available | Not available |
| Akt | Rabbit | Proteintech; 10176-2-AP | 1000 | Not available | Not available |
| p-Akt | Rabbit | Proteintech; 66444-1-Ig | 1000 | Not available | Not available |
| p65 | Rabbit | Proteintech; 10745-1-AP | 1000 | Not available | Not available |
| p-p65 | Rabbit | Proteintech; 82335-1-RR | 1000 | Not available | Not available |
| β-actin | Rabbit | Proteintech; 20536-1-AP | 2000 | Not available | Not available |
| Hsp70 | Mouse | Abcam; ab2787 | 1000 | Not available | Not available |
| TSG101 | Mouse | Proteintech; 28283-1-AP | 1000 | Not available | Not available |
| CD63 | Mouse | Abcam; ab21286 | 1000 | Not available | Not available |
| PDPN | Mouse | Proteintech; 67432-1-Ig | Not available | 200 | Not available |
| IL-6 | Rabbit | Proteintech; 83747-5-RR | Not available | 200 | Not available |
| Goat Anti-Rabbit IgG H&L (Alexa Fluor® 594) | Goat | Abcam; ab150080 | Not available | 200 | Not available |
| Donkey Anti-Mouse IgG H&L (Alexa Fluor® 488) | Donkey | Abcam; ab150105 | Not available | 200 | Not available |
| APC anti-human CCR3 | Mouse IgG1, κ | Biolegend; 310707 | Not available | Not available | 200 |
| FITC anti-human PDPN | Rat IgG2b, κ | Biolegend; 337025 | Not available | Not available | 200 |
| PE anti-human CCR3 | Mouse IgG1, κ | Biolegend; 310705 | Not available | Not available | 200 |
| APC Mouse IgG1, κ Isotype Ctrl | Mouse IgG1, κ | Biolegend; 400119 | Not available | Not available | 1000 |
| FITC Rat IgG2b, κ Isotype Ctrl | Rat IgG2b, κ | Biolegend; 400605 | Not available | Not available | 1000 |
| PE Mouse IgG1, κ Isotype Ctrl | Mouse IgG1, κ | Biolegend; 400112 | Not available | Not available | 1000 |
| Goat Anti-Rabbit IgG H&L (HRP) | Goat | Abcam; ab97051 | 5000 | Not available | Not available |
| Goat Anti-Mouse IgG H&L (HRP) | Goat | Abcam; ab6789 | 5000 | Not available | Not available |
